# Clinical features and surgical options of obstructed hemivagina and ipsilateral renal agenesis (OHVIRA) syndrome: A systematic review and a meta‐analysis of prevalence

**DOI:** 10.1002/ijgo.70164

**Published:** 2025-04-17

**Authors:** Emma Bonetti, Gloria Anderson, Simona Duranti, Federico Ferrari, Franco Odicino, Antonia Testa, Francesco Fanfani, Giovanni Scambia, Ursula Catena

**Affiliations:** ^1^ Department of Clinical and Experimental Sciences University of Brescia Brescia Italy; ^2^ Scientific Directorate Fondazione Policlinico Universitario A. Gemelli, IRCCS Rome Italy; ^3^ Università Cattolica del Sacro Cuore Rome Italy; ^4^ Department of Woman and Child Health and Public Health Fondazione Policlinico Universitario A. Gemelli, IRCCS Rome Italy

**Keywords:** Herlyn–Werner–Wunderlich syndrome, obstructed hemivagina, OHVIRA syndrome, vaginal septum resection

## Abstract

**Background:**

OHVIRA syndrome, a urogenital malformation, lacks standardized management. Narrative reviews exist, but there is no a comprehensive meta‐analysis.

**Objectives:**

The aim of this first systematic review and meta‐analysis is to evaluate the current literature and inform management strategies.

**Search Strategy:**

We searched Scopus, Medline, Embase and Web of Science, up to March 2024.

**Selection Criteria:**

Case series, case reviews, and longitudinal studies on patients with OHVIRA syndrome.

**Data Collection and Analysis:**

Data were extracted and meta‐analyzed using R software.

**Main Results:**

In all, 35 studies (1988–2022) with 526 patients were included. Average symptom onset was 14.45 years, and diagnosis at 16.36 years suggests potential delays. The most common symptoms were abdominal pain (67%, 95% CI 54–77, *I* ^2^ = 66%) and dysmenorrhea (64%, 95% CI 55–72, *I* ^2^ = 46%). Ultrasound (86%, 95% CI 76–92, *I* ^2^ = 58%) and pelvic magnetic resonance imaging (61%, 95% CI 46–74, *I* ^2^ = 72%) were primary imaging modalities. Hematocolpos (55%, 95% CI 42–67, *I* ^2^ = 53%) and hematometra (53%, 95% CI 37–69, *I* ^2^ = 70%) were frequent findings. Pelvic endometriosis, a major long‐term complication, affected 20% of patients. Vaginal septum resection (83%, 95% CI 75–89, *I* ^2^ = 48%) was the most common surgical treatment, often accompanied by laparoscopic endometriosis excision. Hysteroscopic septum resection emerged as a minimally invasive option (78%, 95% CI 46–93, *I* ^2^ = 54%), with high success rate.

**Conclusions:**

Early diagnosis and surgery are crucial to prevent complications. Vaginal septum resection remains the gold standard, while hysteroscopy offers a promising minimally invasive alternative.

## 1 INTRODUCTION

### 1.1 Background

Obstructed hemivagina and ipsilateral renal agenesis (OHVIRA) syndrome, also known as Herlyn–Werner–Wunderlich syndrome, is a congenital malformation of the female urogenital tract.^1^ It results from abnormal Müllerian duct development, first described by Purslow in 1922.^2^ This condition is characterized by the triad of a double uterus, obstructed hemivagina, and ipsilateral renal agenesis, with reported prevalence ranging from 0.1% to 3.8%.^3^ Typically diagnosed during adolescence or young adulthood, OHVIRA presents with non‐specific symptoms like dysmenorrhea, menstrual irregularities, and pelvic pain. These symptoms, often accompanied by a palpable pelvic mass due to hematometra or hematocolpos, can delay diagnosis. Ultrasound and magnetic resonance imaging are the primary imaging modalities used for diagnosis.^4^ Conservative surgical management, involving resection of the obstructive vaginal septum, is the primary treatment to alleviate symptoms and prevent long‐term complications like endometriosis, pelvic infections, and infertility. Early diagnosis and intervention are crucial to optimize outcomes.^5^ Demolitive surgery is reserved for severe, complicated cases or those with recurrent issues.^6^

### 1.2 Objectives

Despite existing narrative reviews, a comprehensive meta‐analysis on the prevalence of surgical management is lacking. The absence of standardized criteria for clinical characteristics, treatment approaches, and outcomes makes it challenging to identify the most effective treatment for patients with OHVIRA syndrome. To address this gap, we conducted the first systematic review and meta‐analysis of prevalence to evaluate the current literature and provide valuable insights that will inform surgical management strategies for this condition.

## 2 METHODS

### 2.1 Eligibility criteria

We included the following types of studies: (1) case series, case reviews, or longitudinal studies describing at least two patients with OHVIRA syndrome; (2) studies that clearly reported the surgical management of OHVIRA syndrome; (3) studies involving patients diagnosed with OHVIRA syndrome after the onset of menstruation; (4) studies providing data on the prevalence of signs, symptoms, and treatments in patients with OHVIRA syndrome; (5) studies published in English or Italian; and (6) studies involving patients with a confirmed diagnosis of OHVIRA syndrome as defined by Herlyn and Werner.^1^ For studies that included both premenarchal and postmenarchal patients, we only considered the postmenarchal patient sample if at least 30% of the cohort were postmenarchal and data were disaggregated. We excluded the following: reviews, abstracts, video articles, case reports with fewer than two patients, and studies focusing on the treatment of OHVIRA syndrome in the early years of life. To ensure a comprehensive understanding of available evidence on surgical treatments for OVHIRA syndrome, we included gray literature. Additionally, a snowball search was performed to capture all relevant articles.

### 2.2 Information sources

Electronic databases, including Scopus, Medline, Embase and Web of Science, were independently screened by two experienced reviewers (EB, GA) using Zotero references manager (v.6.0.37).

### 2.3 Search strategy

The databases were searched from their inception up to March 30, 2024. In cases of disagreement regarding eligibility, a senior colleague (UC) was consulted. The search strategy employed a combination of MeSH terms and keywords, with appropriate Boolean operators, as shown in File S1.

### 2.4 Study selection

This systematic review and meta‐analysis were conducted following PRISMA, Cochrane, and MOOSE guidelines.^7^, ^8^, ^9^ The study protocol was registered in PROSPERO (CRD42024524558). Titles and abstracts were screened by two reviewers (EB, GA), and full texts of potentially relevant studies were retrieved and assessed for eligibility. Discrepancies were resolved through consultation with a senior reviewer (UC).

### 2.5 Data extraction

Two authors (EB, GA) independently extracted data from the included using a standardized table, custom‐built on Excel. A third author (UC) reviewed the data extraction to ensure completeness and accuracy. If an article reported duplicate information from the same patient, the data from both reports were combined to obtain comprehensive information, but only counted as a single case. Observational studies that provided the overall proportion of symptoms, signs, diagnostic tests, and surgical treatment of the OHVIRA patients were included for quantitative synthesis (meta‐analysis). The extracted data items included author, country, year, study design, aim, characteristic of the study participants, clinical features, imaging, surgical treatments, and outcomes. This structured approach ensured a thorough and accurate compilation of relevant information for the meta‐analysis.

### 2.6 Assessment of risk of bias

Two reviews (EB, GA) independently assessed the quality of the included studies using the Joanna Briggs Institute checklists.^10^ Subsequently, 25% of the rating judgments were independently verified by a third author (UC). Studies were graded following the method outlined by Melo et al.^11^ based on the following criteria: (1) low risk of bias—more than a 70% “yes” score; (2) moderate risk of bias—“yes” responses between 50% and 69%; and (3) high risk of bias—“yes” responses were less than 49%. To evaluate publication bias, funnel plots were generated for visual assessment, and Egger's test for funnel plot asymmetry was conducted formally.^12^

### 2.7 Data synthesis

All data reported as median and interquartile range were converted to mean and standard deviation (SD) following the guidelines outlined in the Cochrane Handbook of Systematic Reviews of Interventions (section 7.7.3.5). Categorial variables were summarized using percentages, while continuous variables were described using means and standard deviations (SDs). Given the nature of the studies included in this review, a meta‐analysis of prevalence was conducted.^13^ To summarize the weighted effect size for each study grouping variable, we calculated a pooled prevalence and its 95% confidence interval (CI) using a random effects model. A random effects model was chosen due to the anticipated high clinical, methodological, and statistical heterogeneity among the included studies. For each outcome of interest (e.g. dysmenorrhea), the proportion of cases out of the total number of cases was meta‐analyzed using the R function *metaprop*. For continuous variables, such as mean age, raw weighted means and their 95% CIs were reported using the R function *metamean*. Measures of heterogeneity, including the *I* ^2^ index and the tau‐squared test, were estimated and reported for both pooled prevalences and pooled means. Anticipating high heterogeneity in the results,^14^ we retained the pooled overall estimates and additionally estimated prediction intervals for all outcomes of interest. Prediction intervals provide the expected range of true effects in similar future studies, offering a more comprehensive understanding of the variability across.^15^ All statistical analyses were performed using R software, ensuring robust and reproducible results.

## 3 RESULTS

### 3.1 Study selection

**FIGURE 1:**
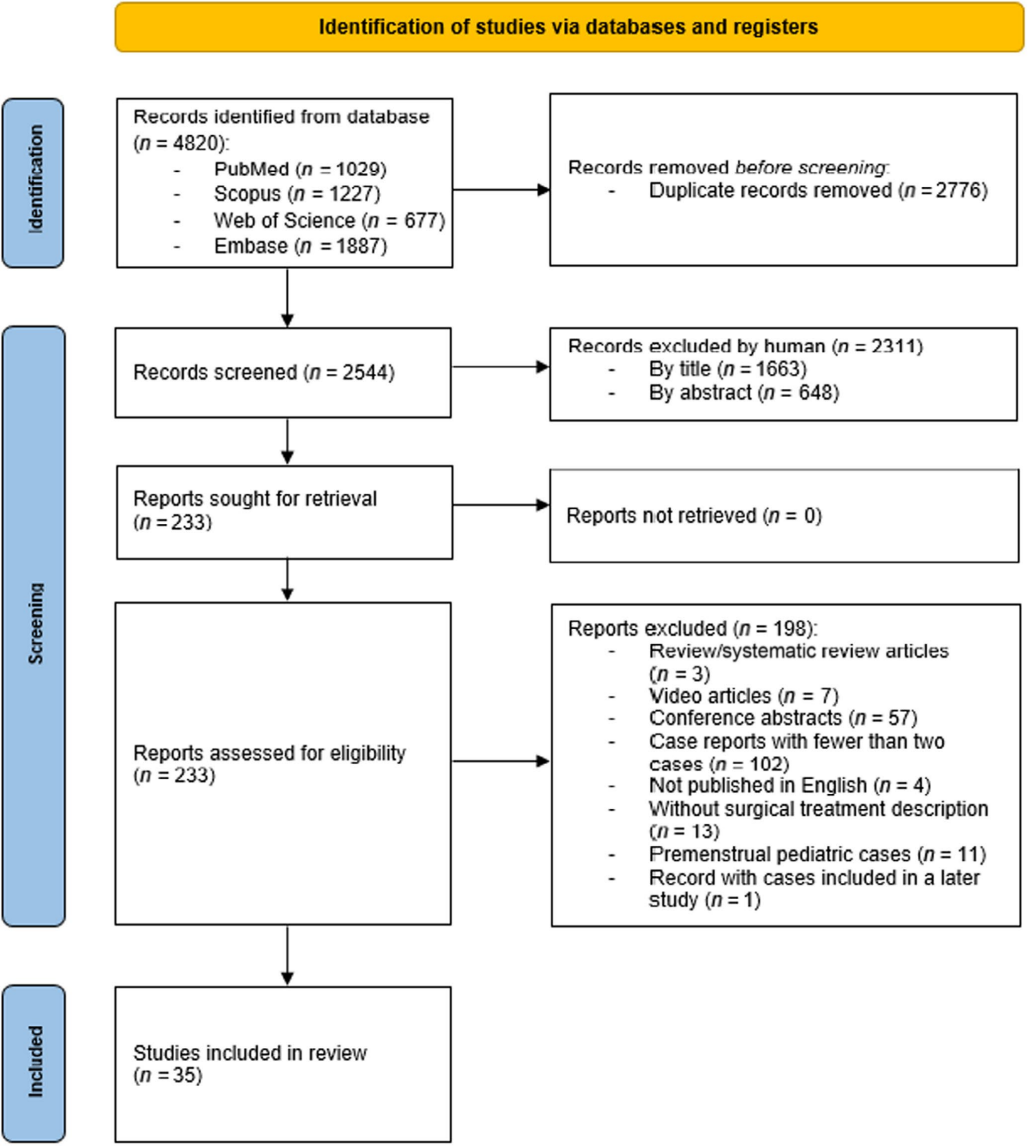
PRISMA flowchart.

The literature search identified a total of 4820 publications. After removing 2776 duplicate records, 2044 unique articles remained. Screening of titles and abstracts led to the exclusion of 2311 records. The full texts of the remaining 233 reports were then assessed for eligibility. Of these, 197 were excluded for not meeting the inclusion criteria, and one additional record was excluded because it presented cases that were later included in a subsequently published study by the same author. Following the selection process, a total of 35 studies were deemed eligible for both qualitative and quantitative synthesis and were included for analysis, as illustrated in Figure 1.^3^, ^4^, ^6^, ^16^, ^17^, ^18^, ^19^, ^20^, ^21^, ^22^, ^23^, ^24^, ^25^, ^26^, ^27^, ^28^, ^29^, ^30^, ^31^, ^32^, ^33^, ^34^, ^35^, ^36^, ^37^, ^38^, ^39^, ^40^, ^41^, ^42^, ^43^, ^44^, ^45^, ^46^, ^47^ No additional studies were identified through the snowball search.

### 3.2 Study characteristics

The included studies were published between 1988 and 2022, with the majority (*n* = 22, 63%) released in the last 10 years, as shown in Table 1. Geographically, most case were conducted in Asia (*n* = 15, 43%), particularly in China (*n* = 6) and Turkey (*n* = 4). Europe accounted for 26% of the studies (*n* = 9), while North America represented 20% (*n* = 7). Patient sample sizes ranged from 2 to 70, with a total of 526 patients analyzed. Nearly half of the studies (*n* = 16, 46%) reported variants of OHVIRA syndrome, including gynecological (*n* = 7) and urological (*n* = 11) anomalies. Interestingly, five studies utilized a validate classification system to categorize patients. Three of these^18^, ^20^ employed the Rock and Jones classification,^48^ which divides patients into three groups: (1) absence of communication with the contralateral Müllerian axis; (2) complete imperforate, with isthmic communication between the two hemiuteri; (3) with incomplete obstruction and no communication between the two hemiuteri. The remaining two studies^44^, ^45^ used a Chinese classification system reported by Bian et al.,^49^ which also categorized patients into three similar groups. Among the 171 patients analyzed in these five studies, the most prevalent subtype was group 1 (*n* = 91, 53.2%), characterized by the absence of communication with the contralateral Müllerian axis.

**TABLE 1:** Overview of the included studies.

Authors	Year of publi‐cation	Country	Study	*N*	Case selection time range	OHVIRA variants	Classification	Risk of bias
Altintas et al.^16^	1998	Turkey	CR	8	1990–1995	8 OHVIRA classic	NR	Low
Bolonduro et al.^6^	2015	USA	CS	5	2012–2014	5 OHVIRA classic	NR	Low
Boudhrâa et al.^17^	2007	Tunisia	CS	4	2000–2004	3 OHVIRA classic 1 ipsilateral ectopic kidney^a^	NR	Low
Candiani et al.^18^	1997	Italy	CR	36	1962–1992	36 OHVIRA classic	Rock and Jones classification: 21 type I 9 type II 6 type III	Low
Candiani et al.^19^	2022	Italy	CR	51	2008–2021	48 OHVIRA classic 1 ureteral remnant opened in the obstructed hemivagina^a^ 2 ipsilateral dysplastic kidneys^a^	Rock and Jones classification: 33 type I 10 type II 8 type III	Low
Cheng et al.^20^	2019	China	CR	14	2009–2017	12 OHVIRA classic 2 septate uteri^b^	Rock and Jones classification: 7 type I 6 type II 1 type III	Low
Da et al.^21^	2014	Malaysia	CS	3	NR	1 OHVIRA classic 1 contralateral renal agenesis^a^ 1 hypoplastic contralateral cervix^b^	NR	Low
Dogan et al.^22^	2016	Turkey	CS	2	NR	2 OHVIRA classic	NR	Low
Fascilla et al.^4^	2020	Italy	CR	5	NR	5 OHVIRA classic	NR	Low
Ghirardo et el.^23^	2008	Italy	CS	2	NR	2 OHVIRA classic	NR	Low
Gholoum et al.^24^	2006	Canada	CR	12	1982–2004	11 OHVIRA classic 1 hypoplastic cervix^b^	NR	Low
Gündüz et al.^25^	2021	Turkey	CS	3	2013–2019	3 OHVIRA classic	NR	Low
Gungor et al.^26^	2020	Turkey	CR	32	2011–2018	31 OHVIRA classic 1 dysplastic kidney^a^	NR	Low
Jha et al.^27^	2020	UK	CS	3	NR	3 OHVIRA classic	NR	Moderate
Kim et al.^28^	2021	South Korea	CR	21	2004–2018	12 OHVIRA classic 4 ipsilateral multicystic kidneys^a^ 5 ectopic ureters^a^	NR	Low
Kimble et al.^29^	2009	Australia	CR	4	2005–2009	3 OHVIRA classic 1 absent ipsilateral ovary and tube^b^	NR	Low
Kozlowski et al.^30^	2020	Poland	CS	2	NR	2 OHVIRA classic	NR	Low
Kriplani et al.^31^	2019	India	CR	8	2012–2018	8 OHVIRA classic	NR	Low
Kudela et al.^3^	2021	Poland	CR	10	2016–2020	10 OHVIRA classic	NR	Low
Lim et al.^32^	2023	Malaysia	CR	18	2013–2022	17 OHVIRA classic 1 dysplastic kidney^a^	NR	Low
Orazi et al.^33^	2007	Italy	CR	11	2000–2006	10 OHVIRA classic 2 ectopic ureteras^a^	NR	Low
Panwar et al.^34^	2021	India	CS	2	NR	2 OHVIRA classic	NR	Low
Piazza et al.^35^	2015	Brazil	CR	19	1984–2009	19 OHVIRA classic	NR	Low
Schlomer et al.^36^	2015	USA	CR	3	2010–2013	3 dysplastic kidneys with ectopic ureters to the obstructed hemivagina^a^	NR	Low
Schutt et al.^37^	2012	USA	CS	3	NR	3 OHVIRA classic	NR	Low
Smith et al.^38^	2007	USA	CR	27	1992–2004	23 OHVIRA classic 3 ipsilateral renal anomalies^a^ 1 contralateral renal anomaly^a^	NR	Low
Stassart et al.^39^	1992	USA	CR	15	1953–1991	15 OHVIRA classic	NR	Low
Tan et al.^40^	2014	China	CR	4	2010–2012	3 OHVIRA classic 1 septate uterus^b^	NR	Moderate
Tong et al.^41^	2013	China	CR	70	1995–2010	70 OHVIRA classic	NR	Low
Tridenti et al.^42^	1988	Italy	CS	3	1979–1986	3 OHVIRA classic	NR	Moderate
Troncon et al.^43^	2018	Brazil	CR	14	1994–2015	14 OHVIRA classic	NR	Low
Wang et al.^44^	2014	China	CR	61	1985–2009	61 OHVIRA classic	Chinese classification: 25 type I 28 type II 8 type III	Low
Yi et al.^45^	2021	China	CR	17	2007–2019	17 septate uteri^b^	Chinese classification: 5 type I 11 type II 1 type III	Low
Zhang et al.^46^	2020	China	CR	26	2009–2017	19 OHVIRA classic 4 septate uteri 1 cervical atresia^b^ 2 ipsilateral renal anomalies with ectopic ureteric orifice into vagina^a^	NR	Low
Zurawin et al.^47^	2004	USA	CR	8	2000–2003	5 OHVIRA classic 3 contralateral renal anomalies^a^	NR	Low

Abbreviations: CS, case series; CR, case review; NR, not reported; OHVIRA, obstructed hemivagina and ipsilateral renal agenesis.

^a^ These cases address urological variations.

^b^ These cases address gynecological variations.

### 3.3 Risk of bias of included studies

Overall, the risk of bias was low, with clear reporting of demographic characteristics, outcomes, and data analysis (Table 1). Additionally, the Egger's test did not suggest the presence of funnel plot asymmetry (*P* = 0.4, File S2).

### 3.4 Synthesis of results

#### Demographic and clinical features

The pooled mean age of menarche was 12.4 years (*k* = 12, 95% CI 12.1–12.6), with a prediction interval ranging from 11.5 to 13.2 years as shown in Table 2. The pooled mean age of symptom onset was 14.45 years (*k* = 14, 95% CI 13–15), although substantial heterogeneity was observed among the studies (*I* ^2^ = 85%, *P* < 0.001). The prediction interval for symptom onset ranged from 10 to 18 years. The pooled mean age at diagnosis 16.36 years (*k* = 17, 95% CI 15.0–17.63), while the pooled mean age at surgery was slightly higher (16.88, 95% CI 13.71–20.05). Given the pooled mean age of the patients at diagnosis, a substantial proportion of patients (65%, 95% CI 46–81) were not sexually active at the time of diagnosis. The most common symptoms at diagnosis were abdominal pain (*k =* 28, 67%, 95% CI 54–77) and dysmenorrhea (*k* = 28, 64%, 95% CI 55–72). Other symptoms included urinary problems (*k* = 9, 32%, 95% CI 10–66), vaginal discharges (*k* = 13, 33%, 95% CI 26–41), and abnormal uterine bleeding (*k* = 17, 20%, 95% CI 17–27). Although dyspareunia appeared to be a prevalent symptom (46%, 95% CI 3–95) in women with OHVIRA syndrome, only three studies provided this information. Notably, there was no substantial heterogeneity between studies for symptoms such as irregular menses, abnormal uterine bleeding and fever (Table 2).

**TABLE 2:** Meta‐analysis of prevalence.

Variable	No. of studies, *k*	Mean or prevalence (95% CI)	Prediction interval	No. of patients, *n*	*I* ^2^	*t* ^2^	*P*‐value^a^
Age							
Menarche	12	12.40 (12.10–12.60)	11.50–13.20	173	60%	0.13	0.004
Symptoms onset	14	14.45 (13.00–15.00)	10.00–18.00	227	85%	3.12	0.0001
Diagnosis	17	16.36 (15.09–17.63)	11.14–21.58	296	89%	5.57	0.0001
Surgery	5	16.88 (13.71–20.05)	4.78–28.98	97	89%	11.8	0.0001
Sexually active							
No	9	0.65 (0.46–0.81)	0.21–0.93	99	41%	0.5	0.09
Symptoms							
Dysmenorrhea	28	0.64 (0.55–0.72)	0.34–0.86	424	46%	0.33	0.005
Abdominal pain	28	0.67 (0.54–0.77)	0.16–0.95	302	66%	1.17	0.0001
Urinary problems	9	0.32 (0.10–0.66)	0.00–0.98	180	84%	3.71	0.0001
Uterine bleeding	17	0.20 (0.17–0.27)	0.13–0.32	376	13%	0.04	0.29
Irregular menses	5	0.26 (0.16–0.39)	0.11–0.48	58	0%	0	0.85
Vaginal discharge	13	0.33 (0.26–0.41)	0.15–0.58	348	48%	0.17	0.02
Dyspareunia	3	0.46 (0.03–0.95)	0.00–1.00	50	89%	7.35	0.0001
Asymptomatic	6	0.12 (0.07–0.21)	0.05–0.26	94	0%	0	0.75
Infertility	5	0.11 (0.03–0.35)	0.00–0.94	121	72%	1.8	0.007
Fever	7	0.13 (0.07–0.22)	0.05–0.31	127	11%	0.07	0.34
Uterus							
Didelphys/bicorporeal/bicornuate	32	0.92 (0.88–0.95)	0.88–0.95	470	0%	0	0.91
Side							
Right	34	0.60 (0.55–0.64)	0.55–0.64	518	0%	0	0.74
Urological anomalies							
Ipsilateral renal agenesis	34	0.89 (0.86–0.92)	0.85–0.92	523	0%	0	0.64
Ipsilateral renal dysplastic/multicystic	8	0.08 (0.03–0.18)	0.00–0.53	230	57%	0.83	0.02
Compensatory contralateral kidney hypertrophy	6	0.77 (0.33–0.95)	0.01–0.99	72	62%	3	0.02
Signs							
Hematocolpos	21	0.55 (0.42–0.67)	0.18–0.87	252	53%	0.59	0.002
Hematometra	20	0.53 (0.37–0.69)	0.08–0.93	255	70%	1.25	0.0001
Hematosalpinx/piosalpinge/tubo‐ovarian abscess	15	0.23 (0.15–0.34)	0.14–0.35	99	0%	0	0.67
Pyometra/pyocolpo	4	0.20 (0.05–0.52)	0.00–0.98	47	53%	1.2	0.09
Hypoplastic/aplastic cervix	6	0.09 (0.03–0.22)	0.00–0.66	149	50%	0.85	0.07
Endometriosis							
Pelvic	11	0.20 (0.13–0.30)	0.06–0.49	206	40%	0.28	0.07
Endometrioma	12	0.12 (0.08–0.17)	0.07–0.18	299	2%	0.01	0.42
Radiological diagnosis							
US	27	0.86 (0.76–0.92)	0.33–0.98	402	58%	1.39	0.0001
MRI	25	0.61 (0.46–0.74)	0.11–0.95	351	72%	1.39	0.0001
CT	11	0.23 (0.13–0.35)	0.06–0.56	129	30%	0.31	0.15
HSG	5	0.26 (0.08–0.58)	0.00–0.97	94	74%	1.6	0.003
IVP	7	0.37 (0.11–0.75)	0.00–0.99	132	87%	3.47	0.0001
Imaging/palpable pelvic mass							
Yes	21	0.62 (0.46–0.76)	0.11–0.95	299	73%	1.4	0.0001
Diagnostic surgery							
Vaginoscopy	9	0.59 (0.26–0.85)	0.01–0.99	62	67%	2.89	0.002
Laparoscopy	18	0.54 (0.37–0.70)	0.07–0.94	341	78%	1.49	0.001
Both vaginoscopy and laparoscopy	3	0.07 (0.02–0.20)	0.00–0.90	52	0%	0	0.39
Laparotomy	6	0.19 (0.10–0.35)	0.04–0.54	63	15%	0.1	0.31
Hormonal therapy							
Yes	5	0.54 (0.25–0.80)	0.06–0.95	16	18%	0.36	0.3
Surgical treatment							
Vaginal septum incision	8	0.31 (0.14–0.57)	0.03–0.87	53	41%	0.91	0.1
Vaginal septum resection	29	0.83 (0.75–0.89)	0.46–0.96	490	48%	0.68	0.002
Hysteroscopic septum resection	5	0.78 (0.46–0.93)	0.04–0.99	37	54%	1.36	0.06
Laparoscopy/robot (hemi‐hysterectomy)	4	0.14 (0.06–0.31)	0–0.81	77	33%	0.32	0.21
Laparotomy (hemi‐hysterectomy)	8	0.13 (0.07–0.23)	0.02–0.43	156	32%	0.31	0.16
Concomitant treatments							
Salpingectomy	4	0.08 (0.03–0.19)	0.00–0.69	114	34%	0.32	0.2
Oophorectomy/adnexectomy	4	0.08 (0.04–0.16)	0.01–0.40	109	9%	0	0.34
Adhesiolysis	5	0.19 (0.06–0.48)	0.00–0.94	73	58%	1.32	0.04
Dye treatments	9	0.21 (0.13–0.34)	0.04–0.62	220	57%	0.4	0.01
Urological	3	0.16 (0.01–0.72)	0.00–1.00	65	79%	4	0.008
Cervicoplasty	3	0.06 (0.01–0.28)	0.00–1.00	80	54%	1.36	0.11
Surgical complications							
Infections	4	0.11 (0.05–0.23)	0.00–0.66	92	27%	0.21	0.25
Stenosis	12	0.14 (0.07–0.24)	0.02–0.54	258	52%	0.66	0.01
None	16	0.89 (0.82–0.93)	0.81–0.93	193	0%	0	0.67
Outcomes							
Abortion rate	3	0.21 (0.11–0.35)	0.002–0.97	45	0%	0	0.5
Live births	12	0.73 (0.59–0.83)	0.36–0.93	119	38%	0.4	0.08
Symptom resolution	18	0.92 (0.88–0.96)	0.87–0.95	264	0%	0	0.77

Abbreviations: CT, computed tomography; HSG, hysterosalpingography; IVP, intravenous pyelogram; MRI, magnetic resonance imaging; US, ultrasound.

^a^ *P*‐value <0.05 is considered statistically significant.

#### Signs and diagnostic findings

Nearly all patients with OHVIRA syndrome (*k* = 32, 92%, 95% CI 88–95) exhibited a double uterus. In the remaining cases, the uterus was septate. Similarly, ipsilateral renal agenesis was the most frequent renal anomaly, occurring in 89% of cases (95% CI 86–92) across nearly all studies (34 out of 35). However, ipsilateral dysplastic or multicystic kidney was documented in only 8% of cases (*k* = 8, 95% CI 3–18), with substantial heterogeneity observed among the studies (Table 2). Compensatory hypertrophy of the contralateral kidney was prevalent in 77% of patients but was reported in only six studies, four of which were published between 2014 and 2022. This finding also showed substantial heterogeneity (*I* ^2^ = 62%, *P* = 0.02), suggesting significant variation in data collection and reporting methods. The malformation predominantly occurred on the right side (*k* = 34, 60%, 95% CI 55–64) compared with the left one. Hematocolpos and hematometra were the most frequently encountered signs, identified in 55% (*k* = 21, 95% CI 42–67) and 53% (*k* = 20, 95% CI 37–69) of cases, respectively. Both signs exhibited substantial heterogeneity, with their prevalence ranging from nearly one‐third to two‐thirds of cases (Table 2). Hemato‐ or pio‐salpinx was consistently observed in 23% of OHVIRA patients (*k* = 15, 95% CI 15–34, *I* ^2^ = 0%, *P* = 0.67) with an almost overlapping prediction interval (95% CI 14–35). The narrow confidence interval and the absence of significant heterogeneity allow us to confidently conclude that tubal involvement affects approximately one in four patients. The prevalence of pelvic endometriosis was observed in 20% (95% CI 13–30) of patients, while ovarian endometriosis was diagnosed in 12% (95% CI 8–17). These findings align with the pooled prediction interval presented in Table 2 for both pelvic and ovarian endometriosis, reinforcing the potential link between endometriosis and OHVIRA syndrome. Ultrasound was the most frequently employed imaging modality for diagnosing OHVIRA syndrome, used in 86% of cases (95% CI 76–92), followed by pelvic magnetic resonance imaging (MRI; 61%, 95% CI 46–74), hysterosalpingography (26%, 95% CI 8–58), and computed tomography (CT) scans (23%, 95% CI 13–35). Substantial heterogeneity was observed for all imaging modalities except for CT scans (Table 2), likely due to regional differences and varying healthcare practices. The most common surgical procedures used for diagnosis were either laparoscopy (*k* = 18, 54%, 95% CI 37–70) or vaginoscopy (*k* = 9, 59%, 95% CI 26–85). Only three studies reported employing both techniques together (Table 2). Laparotomy, reserved for unclear and complicated situations, was used as a diagnostic surgery in 19% of cases (95% CI 10–35, *I* ^2^ = 15%, *P* = 0.31) across six studies.

#### Medical and surgical treatments

**FIGURE 2:**
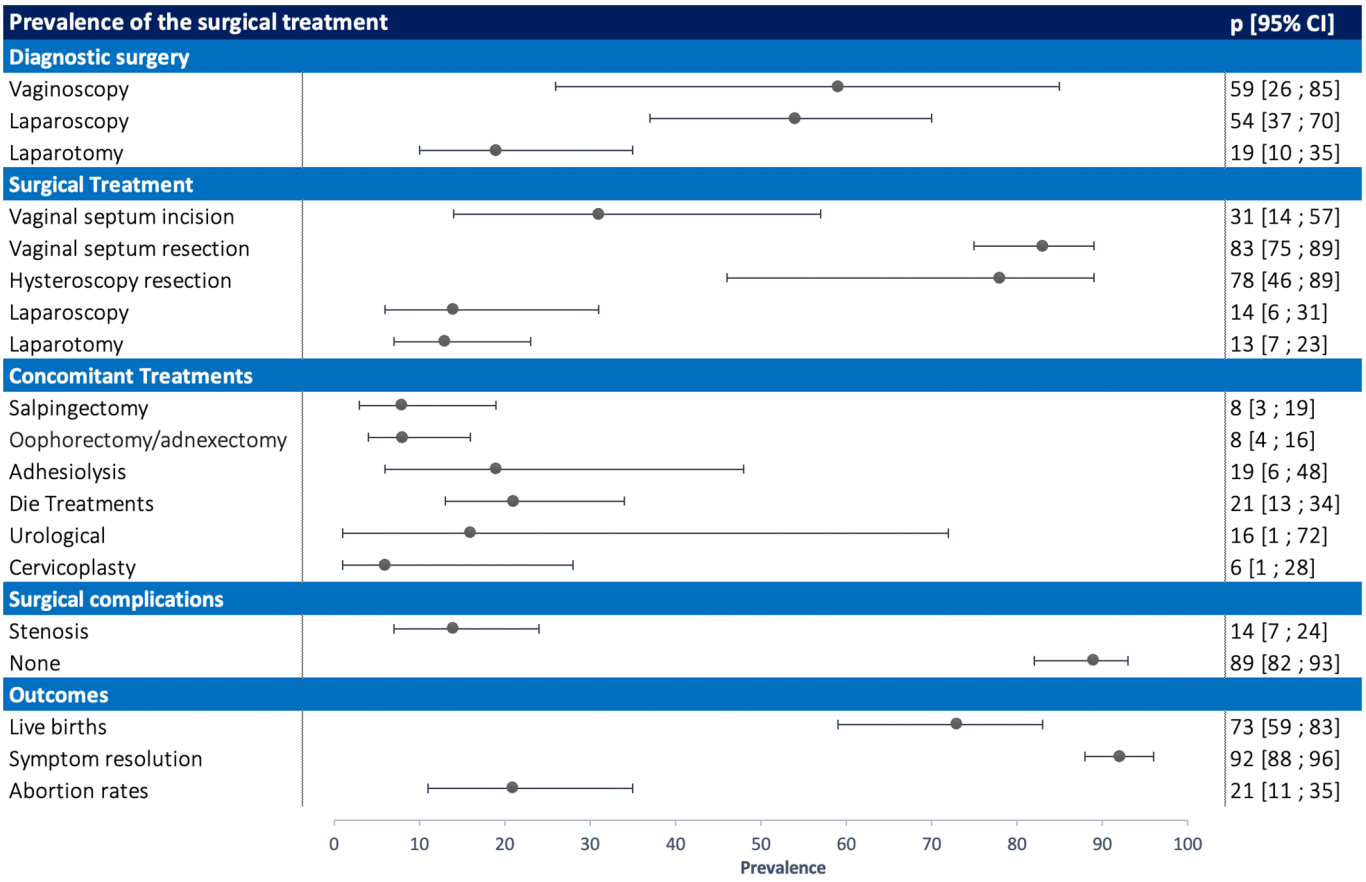
Forest plot of treatments. *P*, prevalence (%). Horizontal axis ranges between 0 and 100.

Only five studies reported the use of preoperative hormonal therapy as shown in Figure 2, with 54% of patients (95% CI 25–80) receiving such treatment. These studies exhibited low heterogeneity (*I* ^2^ = 18%, *P* = 0.36), indicating a consistent trend towards using hormonal therapy. The primary surgical treatments for OHVIRA syndrome focus on the removal of the vaginal septum. Among these procedures, vaginal septum resection emerged as the most prevalent, being employed in 83% of cases (*k* = 29, 95% CI 75–89). A more recent surgical option is hysteroscopic septum resection, first described in a study published in 2012.^37^ The technique was reported in 78% of cases (95% CI 46–93) with no substantial heterogeneity observed among the studies (*I* ^2^ = 54%, *P* = 0.06). However, the limited number of studies (*k* = 5) resulted in a wide prediction interval (95% CI 4–99). A less common surgical approach is vaginal septum incision, which was employed in 31% of cases (*k* = 8, 95% CI 14–57). This procedure involves creating a simple slit in the septum. Due to the scarcity of studies reporting hysteroscopic septum incision (*k* = 2), a reliable meta‐analysis of prevalence was not feasible. Both vaginal and hysteroscopic septum resection aim to eliminate the vaginal septum, thereby creating a permanent opening for menstrual flow. In contrast, septum incision simply creates a slit, which can potentially close again and lead to a recurrence of the original problem. Interestingly, hemihysterectomy was occasionally reported as a surgical option for OHVIRA patients, performed during both laparoscopy (*k* = 4, 14%, 95% CI 6–31) and laparotomy (*k* = 8, 13%, 95% CI 7–23). The most reported concomitant treatments included endometriosis treatment (*k* = 9, 21%, 95% CI 13–34), resolution of urological complications (*k* = 3, 16%, 95% CI 1–72) and adhesiolysis (*k* = 5, 19%, 95% CI 6–48). However, there was high heterogeneity among the studies reporting concomitant procedures (Table 2). Additionally, except for endometriosis treatment, the prediction intervals for both the management of urological complications and adhesiolysis encompassed the zero.

### 3.5 Main outcomes

The analysis revealed a high success rate for surgical treatments, with 89% of cases experiencing no reported complications (*k* = 16, 95% CI 82–93). Similarly, symptom resolution was achieved in 92% of patients (*k* = 18, 95% CI 88–96). The most prevalent complication, reported in 12 out of 35 included studies, was the restenosis of the incision made on the vaginal septum. This occurred in 14% of cases (95% CI 7–24). Live birth and abortion rates were commonly reported outcomes. The analysis indicated a live birth rate of 73% (*k* = 12, 95% CI 59–83) and an abortion rate of 21% (*k* = 3, 95% CI 11–35); however, a significant limitation of the included studies was the lack of long‐term follow‐up data. While data on live birth rate and abortion rate were available, information on other long‐term reproductive outcomes were limited and inconsistent, hindering reliable meta‐analysis (Figure 2).

## 4 DISCUSSION

### 4.1 Comparison with existing literature

This systematic review with meta‐analysis provides a comprehensive overview of OHVIRA syndrome, offering valuable insights into its clinical presentation, diagnosis, and treatment. The substantial heterogeneity observed in symptom prevalence, diagnostic approaches, and surgical techniques highlights the need for standardized diagnostic criteria and treatment protocols. While narrative reviews on OHVIRA syndrome exist, this study represents the first meta‐analysis addressing prevalence and management strategies. This comprehensive review also highlights the diverse presentations of OHVIRA syndrome beyond the traditional triad, underscoring the need for thorough diagnostic evaluations to identify all associated anomalies. The existence of multiple classification systems highlights the need for standardization to improve research comparability, diagnostic accuracy, and treatment consistency. Our analysis reinforces the importance of early diagnosis and prompt surgical intervention to prevent long‐term complications, such as endometriosis and infertility. Resection of the obstructive vaginal septum remains the primary treatment for OHVIRA syndrome. In virgin patients, vaginal resection can be particularly challenging due to a tight vaginal opening, often necessitating hymen disruption. In contrast, hysteroscopic resection is less invasive, suitable for outpatient settings, and can potentially preserve the hymen.

### 4.2 Strengths and limitations

A strength of this study is that it is the first meta‐analysis of OHVIRA syndrome. Despite previous narrative reviews,^50^, ^51^ our meta‐analysis enables a quantitative analysis of the available evidence, leading to a more robust understanding of the condition and addressing that significant gap in the literature by providing a comprehensive systematic review and meta‐analysis of prevalence of this condition.

However, the rarity of OHVIRA syndrome poses a limitation in terms of long‐term follow‐up data on menstrual function, fertility, and pregnancy outcomes. Future research should prioritize long‐term follow‐up studies and the development of standardized diagnostic and treatment protocols to address these limitations and hinder optimal patient care.

### 4.3 Implication

The classic definition of OHVIRA syndrome, characterized by a double uterus, obstructed hemivagina, and ipsilateral renal agenesis, does not capture the full spectrum of presentations identified in our analysis. Of the 35 studies reviewed, 16 reported variations of OHVIRA syndrome, including gynecological and urological anomalies. The most common gynecological variant was a septate uterus, while the most common urological variant involved an ipsilateral dysplastic or multicystic kidney. Given the strong association between Müllerian and renal abnormalities, screening females with Müllerian anomalies for potential renal abnormalities is recommended.^3^ Recognizing these variations can lead to more accurate diagnoses and tailored management strategies, ultimately improving patient outcomes. Our analysis revealed a right‐sided predominance of the malformation, affecting 60% of cases. This finding aligns with previously observed trends in the literature.^52^ The hypothesis that this asymmetry arises before organogenesis warrants further investigation. While a high percentage of cases exhibited compensatory hypertrophy in the contralateral kidney (77%, 95% CI 33–95), the limited number of studies (*n* = 6) and significant heterogeneity in data collection methods necessitate further research to confirm this finding. Classification remains a significant area for standardization. Of the 35 studies reviewed, only a few adopted existing classifications, such as the Rock and Jones^48^ and Bian et al.^49^ classifications. Both systems share three subgroups, with subgroup 1 (absence of communication between the affected hemivagina and the opposite Müllerian axis) being the most prevalent. This subgroup is associated with complications like hematometrocolpos and early symptom onset. Subgroups 2 and 3, with incomplete obstructions, typically present with milder symptoms and later onset. Another classification proposed in 2015 identifies two primary classes—completely and incompletely obstructed hemivaginas—each with two subclasses.^53^ The most common anatomical variants reported are class 1.1 (blind hemivagina) and class 2.1 (partial reabsorption of the vaginal septum), which align with the Rock and Jones classification. A significant delay exists between symptom onset (average age 14.45 years) and diagnosis (average age 16.36 years), which can lead to long‐term complications such as endometriosis, recurrent infections, adhesions, and infertility. Early intervention can reduce the risk of these complications and improve long‐term outcomes, particularly fertility. Endometriosis, a major complication resulting from outflow obstruction and retrograde menstruation, can severely impact fertility.^54^, ^55^ While abdominal pain and dysmenorrhea are the most common presenting symptoms, their non‐specific nature often leads to misdiagnosis. The onset of menarche does not exclude obstructive anomalies, especially in cases of unilateral obstruction. Additionally, pelvic masses resulting from hematometrocolpos can be misdiagnosed as adnexal masses, potentially leading to unnecessary treatments. Heightened clinical awareness and precise diagnostic techniques are crucial to accurately differentiate OHVIRA syndrome from other conditions with similar presentations. Ultrasound and pelvic MRI are the most common diagnostic imaging modalities. Laparoscopy, particularly useful when imaging results are unclear, serves as a diagnostic tool and allows for the simultaneous treatment of coexisting conditions like endometriosis and adhesions. As demonstrated by Kimble et al., a combined approach of vaginal septum resection and laparoscopy can effectively address both the primary condition and any associated pelvic pathologies.^29^ By reducing menstrual bleeding, hormonal therapy can be considered as a temporary measure before surgery, alleviating symptoms and preventing complications. However, long‐term safety and efficacy require further study. Although the vaginal approach has been the most commonly used method, hysteroscopy has emerged as a more recent and promising surgical option, with its first reported use in 2012.^37^ Fascilla et al. demonstrated the feasibility of hysteroscopic incision of the vaginal septum in an office setting without anesthesia, utilizing 5 Fr bipolar electrodes.^4^ Similarly, Cheng et al. reported successful hysteroscopic septum incisions using a “no touch” technique.^20^ Additionally, the accuracy of the procedure can be further enhanced by employing transabdominal or transrectal ultrasound guidance.^56^, ^57^ Our analysis revealed a critical distinction between septum incision and resection. Both vaginal and hysteroscopic resections, which involve complete removal of the vaginal septum, create a permanent opening and minimize the risk of restenosis (14%, 95% CI 7–24). In contrast, septum incision, which only creates a slit, can lead to recurrence. Hemi‐hysterectomy may be considered in complex cases, where vaginoplasty is not feasible, or those with delayed diagnosis leading to severe complications. Most surgical treatments reported high success rates and significant symptom resolution. While the reported live birth rate of 73% is promising, more comprehensive long‐term data are needed.

## 5 CONCLUSION

Obstructed hemivagina and ipsilateral renal agenesis syndrome is a complex condition requiring a multidisciplinary approach for optimal diagnosis and management. This meta‐analysis emphasizes the importance of minimally invasive techniques, particularly hysteroscopy, in surgical intervention. Early diagnosis and treatment are crucial for preventing complications and preserving future fertility. By highlighting the need for early recognition to prevent complications, this meta‐analysis underscores the importance of standardized diagnostic and treatment protocols. Future research should focus on long‐term outcomes to improve patient care and quality of life.

## AUTHOR CONTRIBUTIONS

EB was involved in the conception, planning, literature search, selection of studies, data extraction, data analysis and writing up of the review. GA was involved in the conception, planning, literature search, selection of studies, data extraction, data analysis, meta‐regression analysis, and writing up of the review. SD was involved in the conception, planning, literature search, and selection of studies. FF was involved in the writing up of the review and supervision. FO was involved in supervision and review. AT was involved in supervision and review. FF was involved in supervision and review. GS was involved in supervision and review. UC was involved in the conception, planning, literature search, selection of studies, data extraction, data analysis, writing up of the review, overall supervision, review, and editing.

## CONFLICT OF INTEREST STATEMENT

The authors have no conflicts of interest.

## Supporting information


File S1.

File S2.

## Data Availability

Data sharing is not applicable to this article as no new data were created or analyzed in this study.
