# Supplementary material for: Clinical features and surgical options of obstructed hemivagina and ipsilateral renal agenesis (OHVIRA) syndrome: A systematic review and a meta‐analysis of prevalence
Source: Int J Gynaecol Obstet. 2025 Apr 17;171(1):152–64. doi: 10.1002/ijgo.70164 (PMC12447673; doi:10.1002/ijgo.70164)
Supplement: Supplementary file 1 — File S1. [file IJGO-171-152-s002.docx]

**Pubmed (n = 1029)**

("Ohvira syndrome" OR OHVIRA OR "obstructed hemivagina" OR "obstructed hemi-vagina" OR "blind hemivagina" OR "Herlyn-Werner-Wunderlich syndrome" OR "Herlyn Werner Wunderlich syndrome" OR "didelphys uterus" OR "uterine didelphys" OR "didelphic uterus" OR "bicorporeal uterus" OR "bicornuate uterus" OR "double uterus" OR "obstructed hemivagina and ipsilateral renal anomaly syndrome" OR "Duplicated vagina") AND ("surgical treatment" OR "surgical treatments" OR Surgery OR laparoscopic OR laparoscopy OR LPS OR Vaginoscopy OR vaginoscopic OR Hysteroscopy OR hysteroscopic OR Resectoscope OR Ecography OR Ultrasound OR ultrasounds OR Ultrasonography OR imaging OR Diagnosis OR "minimally invasive endoscopic approach" OR Endoscopic OR "vaginal resection" OR Vaginoplasty OR "Vaginoscopic resection" OR "hysteroscopic septoplasty" OR "minimally invasive surgery" OR "conservative treatment" OR "MRI scan" OR "MRI scans" OR "magnetic resonance" OR "early diagnosis" OR Hemihysterectomy OR Hysterectomy OR "Unilateral hysterectomy" OR management)

**Scopus (n = 1227)**

("Ohvira syndrome" OR OHVIRA OR "obstructed hemivagina" OR "obstructed hemi-vagina" OR "blind hemivagina" OR "Herlyn-Werner-Wunderlich syndrome" OR "Herlyn Werner Wunderlich syndrome" OR "didelphys uterus" OR "uterine didelphys" OR "didelphic uterus" OR "bicorporeal uterus" OR "bicornuate uterus" OR "double uterus" OR "obstructed hemivagina and ipsilateral renal anomaly syndrome" OR "Duplicated vagina") AND ("surgical treatment" OR "surgical treatments" OR Surgery OR laparoscopic OR laparoscopy OR LPS OR Vaginoscopy OR vaginoscopic OR Hysteroscopy OR hysteroscopic OR Resectoscope OR Ecography OR Ultrasound OR ultrasounds OR Ultrasonography OR imaging OR Diagnosis OR "minimally invasive endoscopic approach" OR Endoscopic OR "vaginal resection" OR Vaginoplasty OR "Vaginoscopic resection" OR "hysteroscopic septoplasty" OR "minimally invasive surgery" OR "conservative treatment" OR "MRI scan" OR "MRI scans" OR "magnetic resonance" OR "early diagnosis" OR Hemihysterectomy OR Hysterectomy OR "Unilateral hysterectomy" OR management)

**WoS (n = 677)**

[("osvira syndrome" OR osvira OR "obstructed hemivagina" OR "obstructed hemi-vagina" OR "blind hemivagina" OR "Herlyn-Werner-Wunderlich syndrome" OR "Herlyn Werner Wunderlich syndrome" OR "didelphys uterus" OR "uterine didelphys" OR "didelphic uterus" OR "bicorporeal uterus" OR "bicornuate uterus" OR "double uterus" OR "obstructed hemivagina and ipsilateral renal anomaly syndrome" OR "Duplicated vagina") AND ("surgical treatment" OR "surgical treatments" OR Surgery OR laparoscopic OR laparoscopy OR LPS OR vaginoscopical OR vaginoscopical OR Hysteroscopy OR hysteroscopic OR Resectoscope OR echography OR Ultrasound OR ultrasounds OR Ultrasonography OR imaging OR Diagnosis OR "minimally invasive endoscopic approach" OR Endoscopic OR "vaginal resection" OR Vaginoplasty OR "vaginoscopical resection" OR "hysteroscopic septoplasty" OR "minimally invasive surgery" OR "conservative treatment" OR "MRI scan" OR "MRI scans" OR "magnetic resonance" OR "early diagnosis" OR Hemihysterectomy OR Hysterectomy OR "Unilateral hysterectomy" OR management)](https://www-webofscience-com.proxy.unibs.it/wos/woscc/summary/59c12611-e18d-4c9b-9207-1babe2851e4d-9ef20e82/relevance/1)

**Embase (n = 1887)**

(“Ohvira syndrome” OR OhvIRA OR “obstructed hemivagina” OR “obstructed hemi-vagina” OR “blind hemivagina” OR “Herlyn-Werner-Wunderlich syndrome” OR “Herlyn Werner Wunderlich syndrome” OR “didelphys uterus” OR “uterine didelphys” OR “didelphic uterus” OR “bicorporeal uterus” OR “bicornuate uterus” OR “double uterus” OR “obstructed hemivagina and ipsilateral renal anomaly syndrome” OR “Duplicated vagina”)/br AND ((“surgical treatment” OR “surgical treatments” OR Surgery OR laparoscopic OR laparoscopy OR LPS OR Vaginoscopy OR vaginoscopic OR Hysteroscopy OR hysteroscopic OR Resectoscope OR Ecography OR Ultrasound OR ultrasounds OR Ultrasonography OR imaging OR Diagnosis OR “minimally invasive endoscopic approach” OR Endoscopic OR “vaginal resection” OR Vaginoplasty OR “Vaginoscopic resection” OR “hysteroscopic septoplasty” OR “minimally invasive surgery” OR “conservative treatment” OR “MRI scan” OR “MRI scans” OR “magnetic resonance” OR “early diagnosis” OR Hemihysterectomy OR Hysterectomy OR “Unilateral hysterectomy” OR “Management”):ti,ab,kw)
