# Supplementary figures and images for: Clinical features and surgical options of obstructed hemivagina and ipsilateral renal agenesis (OHVIRA) syndrome: A systematic review and a meta‐analysis of prevalence
Source: Int J Gynaecol Obstet. 2025 Apr 17;171(1):152–64. doi: 10.1002/ijgo.70164 (PMC12447673; doi:10.1002/ijgo.70164)

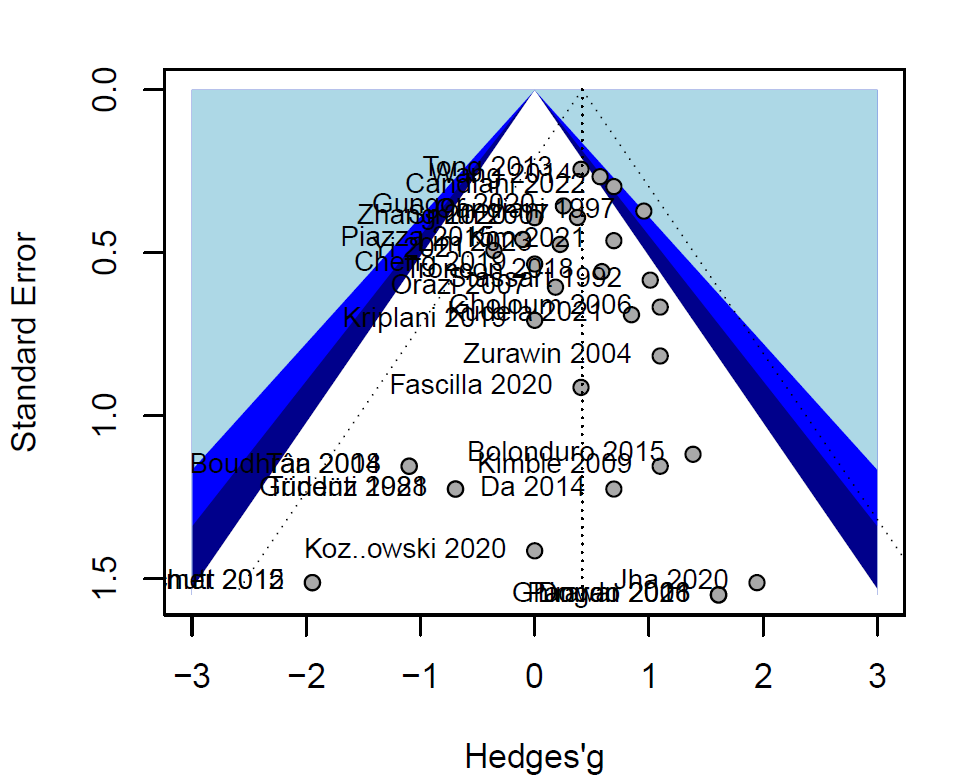

Supplement: Supplementary file 2 — File S2. [file IJGO-171-152-s001.docx]
